# Impact of targeted infection prevention and control interventions on bloodstream infections and colonization with carbapenem-resistant organisms among hospitalized neonates, Bangladesh, 2023–2024

**DOI:** 10.1186/s13756-026-01765-0

**Published:** 2026-05-20

**Authors:** Ashley Styczynski, Amelia Keaton, Gazi Md. Salahuddin Mamun, Sanzida Khan, Shariful Amin Sumon, Tanzir Ahmed Shuvo, Shabrina Sharmin, Md. Aminul Islam, Aninda Rahman, Tahsinul Amin, Jesmin Akter, Kakali Halder, Sazzad Bin Shahid, Gemma Parra, Lisa P. Oakley, Katie Wilson, Molly Patrick, Md Golam Dostogir Harun, Fahmida Chowdhury

**Affiliations:** 1 Division of Healthcare Quality and Promotion, U.S. Centers for Disease Control and Prevention, Atlanta, GA USA; 2 Infectious Diseases Division, International Centre for Diarrhoeal Disease Research, Bangladesh (icddr,b), Dhaka, Bangladesh; 3 Directorate General of Health Services, Ministry of Health and Family Welfare, Dhaka, Bangladesh; 4 Department of Neonatology, Dhaka Medical College Hospital, Dhaka, Bangladesh; 5 Department of Microbiology, Dhaka Medical College Hospital, Dhaka, Bangladesh; 6 Division of Infectious Disease Readiness and Innovation, U.S. Centers for Disease Control and Prevention, Atlanta, GA USA

## Abstract

**Background:**

Neonates admitted to neonatal intensive care units (NICUs) are particularly susceptible to acquiring healthcare-associated infections because of factors such as prematurity, prolonged hospital stays, and need for invasive support devices. A longitudinal study in a Bangladeshi NICU demonstrated a high burden of bloodstream infections (BSIs) and rectal colonization with carbapenem-resistant organisms (CROs). We aimed to assess the impact of infection prevention and control (IPC) interventions on BSIs and CRO colonization in this NICU.

**Methods:**

We conducted a quasi-experimental study in a 38-bed NICU of a tertiary care referral hospital in Dhaka, Bangladesh. Following a baseline assessment in December 2023-January 2024, an IPC intervention was carried out during February-September 2024, with a pause during June-August because of political instability. NICU staff, including environmental cleaners, received multimodal IPC training with an emphasis on hand hygiene and environmental cleaning. Compliance was monitored through covert observation of hand hygiene and use of fluorescent markers on environmental surfaces. Colonization with CROs was evaluated through twice monthly point prevalence surveys using rectal swabs plated on selective media followed by Vitek 2 confirmatory testing. BSIs were assessed based on recovery of pathogenic organisms from blood cultures, and mortality rates were determined based on the number of deaths per 100 admissions. We assessed for trends in process and outcome measures over time using the Mann-Kendall test.

**Results:**

Over the course of the IPC intervention, hygiene compliance improved from 13% to 69% (*p* < 0.001) while environmental cleaning improved from 10% to 87% (*p* < 0.001). We sampled 363 neonates across 12 point prevalence surveys. CRO colonization was similar at the beginning of the intervention compared with the endline (88% vs. 81%, *p* = 0.45). However, BSI incidence decreased from 28 to 4 per 100 admissions (*p* = 0.034), representing an 86% reduction. Similarly, mortality decreased from 26 to 4 per 100 admissions (*p* = 0.064), though this change was not statistically significant.

**Conclusions:**

Enhancing basic IPC practices may be effective in reducing adverse outcomes in low-resource settings with a high burden of multidrug-resistant organisms (MDROs). However, the lack of impact on CRO colonization suggests the need for additional IPC and/or antibiotic stewardship interventions to further mitigate MDRO transmission and the selective pressures driving MDRO proliferation in this NICU.

**Supplementary Information:**

The online version contains supplementary material available at 10.1186/s13756-026-01765-0.

## Background

 Neonates requiring admission to neonatal intensive care units (NICUs) are vulnerable to infections of all kinds due to factors such as immature immune systems, congenital anomalies, need for invasive medical interventions, and prolonged healthcare stays [1–4]. Neonates in low- and middle-income countries (LMICs) are particularly vulnerable to antimicrobial-resistant infections, where healthcare facilities may lack access to basic hygiene services and expanded therapeutic options may not be available [5–11]. Notably, rising antimicrobial resistance in neonates has been identified as a key barrier to reaching the United Nations Sustainable Development Goals for under-5 mortality [12]. 

Asymptomatic colonization with antimicrobial-resistant organisms in neonates is a known precursor to infection in premature and sick neonates, making it an attractive target for reducing infections [13, 14]. Additionally, even infants who never develop clinical infections may still transmit colonizing bacteria to other infants within the same unit. While comprehensive infection prevention and control (IPC) measures such as hand hygiene, environmental cleaning, medical device reprocessing, antimicrobial stewardship, and transmission-based precautions are known to be effective in preventing colonization in older children and adult populations, such successes are typically resource-intensive and often rely on the availability of ongoing colonization screening [15–20]. The effectiveness of such interventions remains unclear in neonatal populations with a high burden of colonization with antimicrobial-resistant bacteria, as colonization may also be influenced by maternal health, the delivery setting, and other factors aside from the neonatal care environment. Moreover, targeted efforts for reducing colonization have mainly focused on gram-positive organisms [21–25]. The effectiveness of existing decolonization strategies for reducing gram-negative colonization and infections is less certain [26–29]. This issue is especially germane for LMICs where neonatal infections are predominantly caused by gram-negative bacteria, including antimicrobial-resistant bacteria [30–32]. 

In this study, we describe the implementation and evaluation of an IPC intervention to reduce colonization and infections caused by multidrug-resistant organisms (MDROs) in a NICU in a resource-limited setting. The study was carried out as a quality improvement initiative in response to findings that over 90% of neonates in this NICU were found to have rectal colonization with MDROs, particularly carbapenem-resistant *Klebsiella pneumoniae*, by the time of discharge (unpublished data, F. Chowdhury, personal communication, December 13, 2023). We define MDROs according to previously published definitions of resistance of an organism to at least one antibiotic in three or more antimicrobial categories, which includes carbapenem-resistant organisms (CROs) [33]. The intervention targeted key drivers of transmission, including hand hygiene compliance and environmental contamination, and was designed for adaptability in settings with constrained infrastructure, training, and staffing. Our objective was to determine whether this pragmatic approach could contribute to improved infection control and reduce the prevalence of MDROs in a vulnerable neonatal population.

## Methods

### NICU characteristics and baseline IPC assessment

The participating NICU is a 38-bed unit at a publicly operated, tertiary care referral hospital in Dhaka, Bangladesh. The NICU typically operates at full bed capacity, with an average of 114 admissions per month, including inborn neonates and neonates transferred from home or other facilities shortly after birth. Prematurity is the most common reason for admission to this NICU followed by low birth weight, neonatal jaundice, congenital heart disease, and birth asphyxia. BSIs among the neonates in this unit are commonly caused by MDROs. The nurse-to-patient ratio ranges from 1:3 to 1:9 depending upon time of day and staffing levels. Environmental cleaners often also assist with patient care activities (such as diaper changes). The unit has an open-bay design, and only one neonate is placed in each crib, though cribs are frequently < 1 m apart due to space constraints.

We first assessed baseline unit IPC practices from December 2023-January 2024 through review of unit policies and repeated observations of unit IPC measures. At baseline, the ability to provide training on IPC practices was considered limited due to healthcare worker (HCW) turnover within the hospital, as well as varying literacy levels among staff cadres; structured training on environmental cleaning was not available. Hand hygiene supplies were available at points of care (waterless hand rub), and one sink was available in the unit with consistent access to running water and soap, though the sink was also close to patient care activities and was utilized for activities other than hand hygiene. Single-use hand towels were often not available. Inconsistent availability of personal protective equipment (PPE) and single-use medical supplies such as feeding equipment and respiratory supplies were also noted, which led to reuse of some of these items.

### IPC intervention and measurements

Based on findings from the baseline assessments and available resources, we focused the interventions on improving environmental cleaning and hand hygiene as these are considered essential elements of infection prevention and are feasible to sustain in a low-resource setting [34]. 

The IPC intervention was implemented during February-September 2024 with a pause during July-August because of political instability. The multimodal intervention consisted of baseline and monthly trainings specific to HCW cadres (physicians, nurses, and environmental cleaners), reminders in the workplace, and system change [35]. Training topics for clinical staff didactics (physicians and nurses) included hand hygiene, waste management, use of personal protective equipment, and prevention of needle stick injuries. This was accompanied by placement of posters in the unit instructing on hand hygiene moments, hand hygiene techniques, and waste management using color-coded bins.

Environmental cleaners also received trainings on hand hygiene as well as on cleaning procedures for high touch and low touch surfaces, cleaning solution preparation, and mopping techniques. Trainings were accompanied by job aids, including an IPC training manual, an infographic on cleaning steps, a cleaning checklist, and a flip chart on cleaning procedures. These materials were designed to be accessible to environmental cleaners with limited literacy, and utilized photos and videos taken within the unit as well as explanatory pictorials drawn by a local artist (Supplemental Fig. 1). Additionally, a hand hygiene station was set up outside the NICU for use by visitors accompanied by posters on proper hand hygiene practices.

Hand hygiene compliance was assessed with covert, direct observation during twice monthly audits conducted for all three categories of HCWs using the WHO 5-moments hand hygiene methodology and observation tool [36]. Observation sessions were conducted at different times of the day and during all shifts. Environmental cleaning compliance was assessed through use of fluorescent markers applied to five patient zone surfaces, five common surfaces, and five surfaces of shared equipment on a twice monthly basis (Supplemental Table 1). The selected surfaces and days of monitoring rotated throughout the intervention so that environmental cleaning staff would not be aware of which surfaces were marked and when.

### Colonization screening, bloodstream infection incidence, and mortality

We conducted point prevalence surveys (PPSs) for rectal colonization with CROs twice monthly during the intervention. We collected rectal swabs from all neonates present on the unit on a specific day for whom parents/guardians provided consent. We plated the swabs on selective media (CHROMagarmSuperCARBA) followed by confirmation of species identification and antibiotic susceptibility using Vitek 2 (bioMérieux, Marcy-l’Étoile, France) for any recovered colonies. We determined colonization prevalence based on the percentage of neonates with Vitek-confirmed CROs recovered from rectal swabs at each PPS. We collected limited clinical information from the patient charts at the time of the colonization screening for the purposes of contextualizing the findings, though we did not collect any patient identifiers or conduct any longitudinal follow-up.

We obtained data on incident bloodstream infections (BSIs) and deaths based on chart reports and aggregate ward registries. Blood cultures were performed for neonates with suspected sepsis per clinician discretion. Blood cultures were processed at either of two labs – one employed manual methods and the other used automated methods – per the clinician discretion. From PPS 8 onwards, all blood cultures were performed using manual methods. We defined BSI as having a positive blood culture with organisms other than common commensals (i.e., coagulase-negative *Staphylococcus* and *Bacillus* spp.) [37]. We calculated BSI incidence as the number of positive blood cultures compared with the number of admissions in each sampling period. We determined mortality rate based on the number of deaths per 100 admissions in a sampling period. We assessed changes in process measures and outcome metrics using the Mann-Kendall test for trends.

## Results

### Neonatal clinical characteristics

We sampled 363 neonates over the course of the intervention period, with an average of 30 (range: 23–38) neonates included in each PPS. Although not all neonates in the unit participated in each PPS, those who were enrolled represented over 95% of the neonates in the unit. The median age of the neonates was 10 (IQR 6–19) days, and the median duration of NICU stay prior to sample collection was 6 (IQR 2–10) days (Table 1). Most of the neonates (62.8%) had been transferred from another hospital, and the majority (58.4%) had been delivered by cesarean section. Of the enrolled neonates, 44.1% had a clinical diagnosis of sepsis at the time of sampling. All neonates with available information were receiving antibiotics (prophylaxis or treatment).

**Table 1:** Clinical characteristics of neonates included in cross-sectional colonization screening from a NICU in Bangladesh, Feb–Sept 2024

Clinical characteristics (*N* = 363)	*N* (%) or median (IQR)
Age in days	10 (6–19)
NICU stay prior to sample collection (days) (median, IQR)	6 (2–10)
Sex (male)	227 (62.5%)
Delivery by cesarean section	212 (58.4%)
Preterm (*n* = 360)	210 (58.3%)
Low birth weight (*n* = 362)	231 (63.8%)
Admitted from	
Home	47 (13.0%)
Delivery ward	88 (24.2%)
Other hospital	228 (62.8%)
Clinical sepsis (at time of sampling)	160 (44.1%)
Antibiotic administration (*n* = 346)	346 (100%)
Central venous catheter	23 (6.3%)
Mechanical ventilation	16 (4.4%)
Feeding modalities*	
Breast milk	100 (27.5%)
Nasogastric/orogastric tube feeding	88 (24.2%)
Intravenous (IV) fluid	220 (60.6%)

*Not mutually exclusive; NICU = neonatal intensive care unit, IQR = interquartile range

*IPC assessments and intervention*

**Fig. 1:**
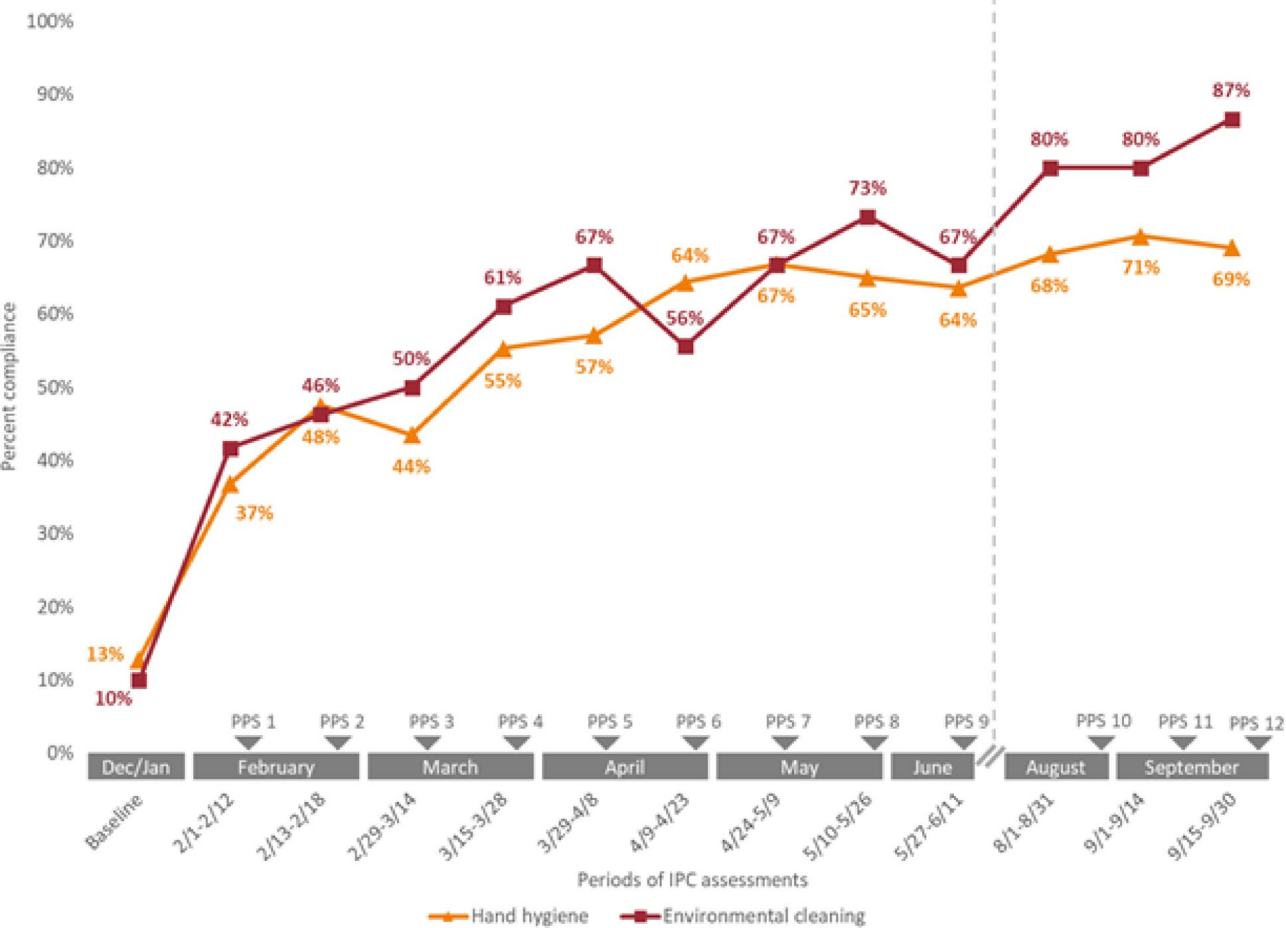
Hand hygiene and environmental cleaning compliance during an infection prevention and control intervention in a NICU in Bangladesh, December 2023-September 2024. *PPS = point prevalence survey. Dates indicate the period of surveillance preceding each PPS. Vertical dashed line indicates a pause in activities during a period of political instability (July–August 2024).*

Initial IPC assessments in December 2023-January 2024 demonstrated 13% compliance with hand hygiene across the WHO five moments and 10% compliance with environmental cleaning (Fig. 1, Supplemental Table 1). Continual improvements in these metrics were observed over the course of the intervention, increasing to 69% hand hygiene compliance (*p* < 0.001) and 87% environmental cleaning compliance (*p* < 0.001) at the last data collection point. Although the intervention concluded in September, additional observations through December 2024 showed persistent improvements with 70% hand hygiene compliance and 83% environmental cleaning compliance

### Colonization screening, BSI incidence, and mortality

**Fig. 2:**
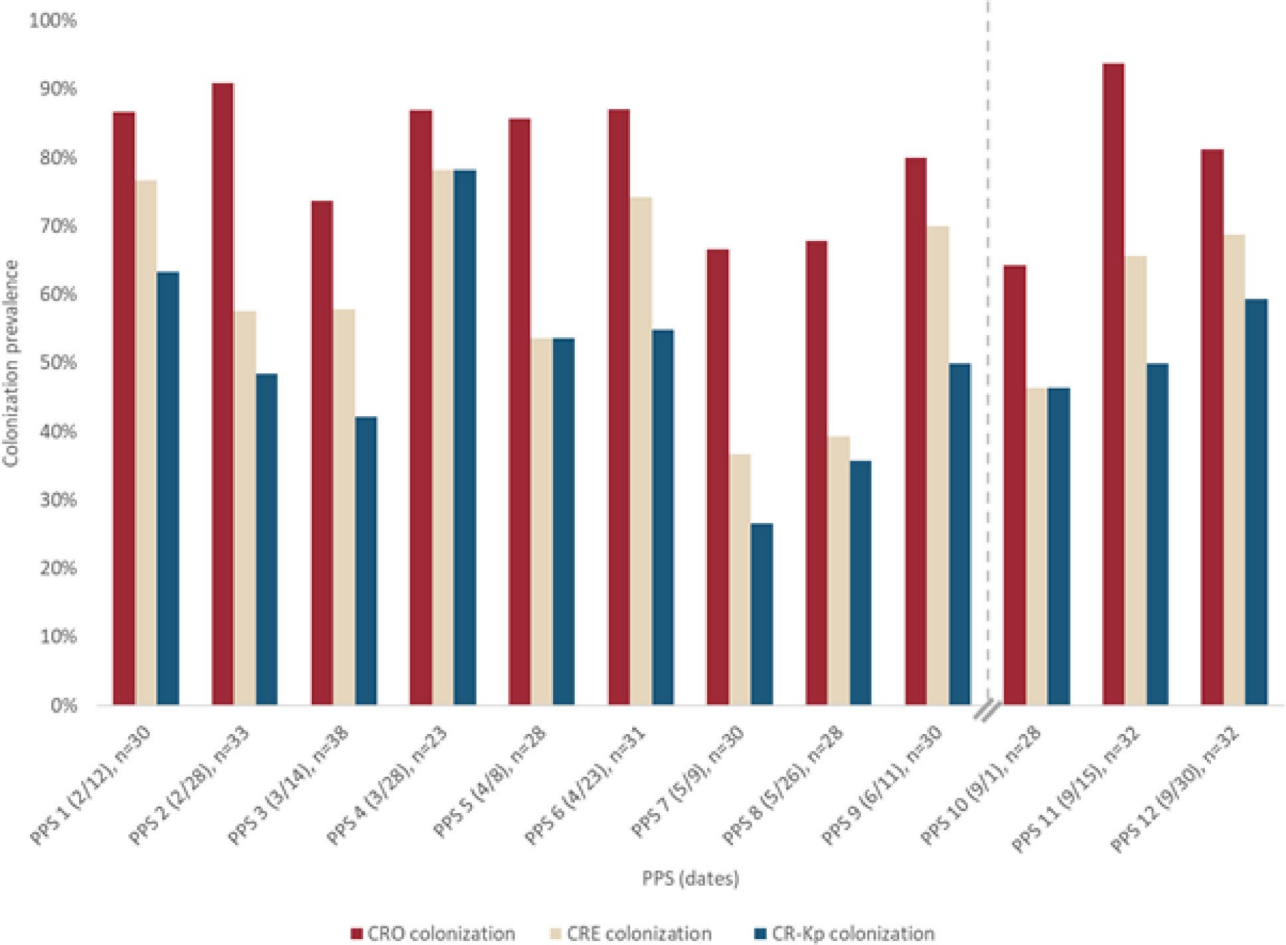
Colonization prevalence with CRO, CRE, and CR-Kp among neonates admitted to a NICU in Bangladesh, February-September 2024. PPS = point prevalence survey, CRO = carbapenem-resistant organism, CRE = carbapenem-resistant Enterobacterales, CR-Kp = carbapenem-resistant *K. pneumoniae. Vertical dashed line indicates a pause in activities during a period of political instability (July–August 2024).*

Colonization prevalence from PPS 1 was comparable to the prior longitudinal data with 87% of neonates colonized with CROs. The majority (23/26, 88%) of CROs were found to be Enterobacterales (CRE), which were primarily *K. pneumoniae* (CR-Kp) (19/23, 83%). CRO, CRE, and CR-Kp colonization did not significantly change over the course of the IPC intervention (CRO *p* = 0.45, CRE *p* = 0.49, CR-Kp *p* = 0.78) (Fig. 2, Supplemental Table 2). By the final PPS, CRO colonization remained at 81% with CR-Kp colonization at 59%.

**Fig. 3:**
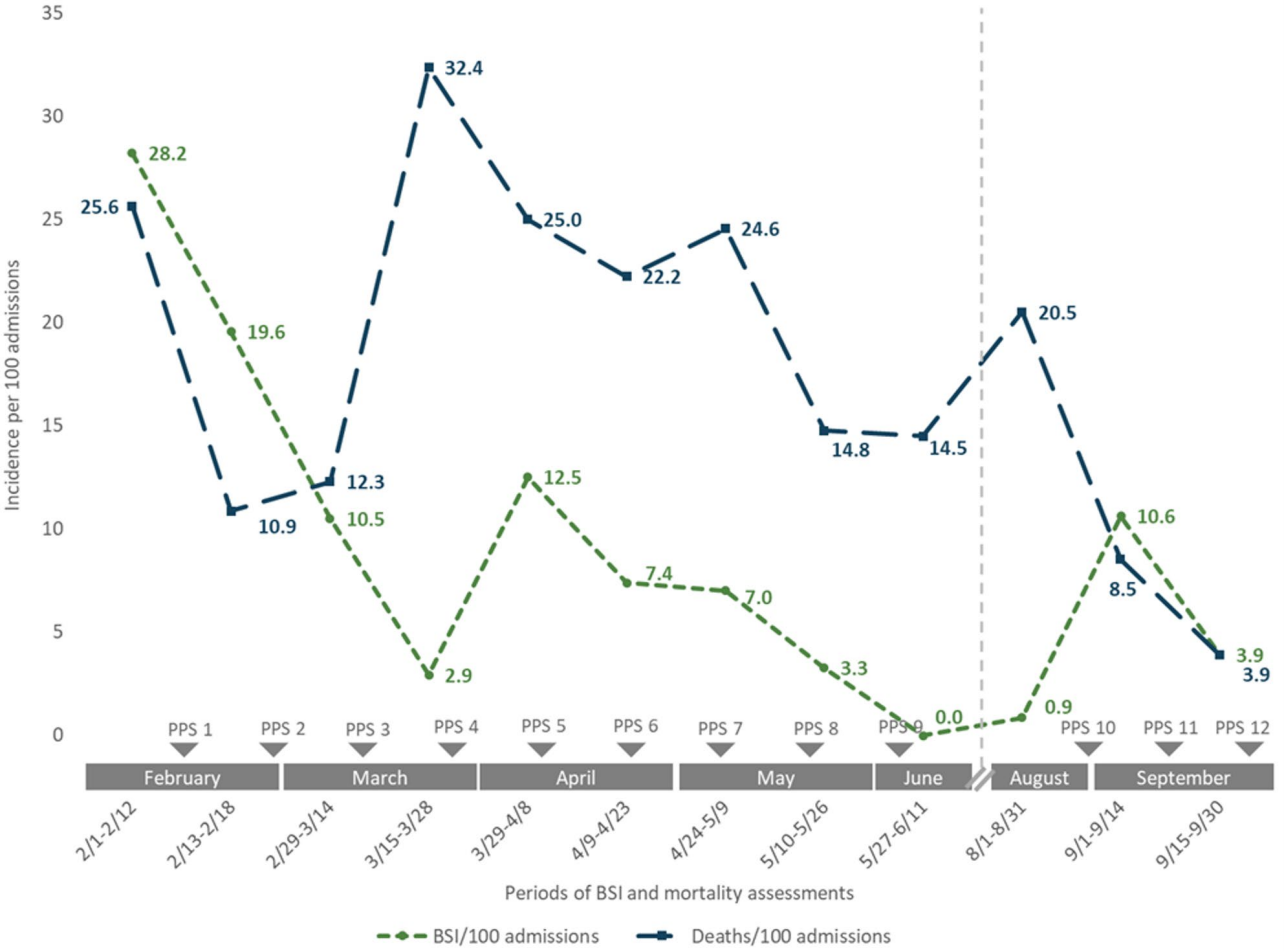
Incidence of bloodstream infections and mortality among neonates admitted to a NICU in Bangladesh, December 2023-September 2024. *PPS = point prevalence survey. Dates indicate the period of surveillance preceding each PPS. BSI = bloodstream infection. Vertical dashed line indicates a pause in activities during a period of political instability (July–August 2024).*

In the current study, for the two weeks preceding the first PPS, BSI incidence was found to be 28.2 per 100 admissions. Substantial variability was seen between PPS time points but with an overall downward trend over the course of the intervention (*p* = 0.034) (Fig. 3). BSI incidence decreased to 3.9 per 100 admissions by the end of the intervention period, representing an 86% reduction. The causative etiologies of the BSIs are listed in Supplemental Table 3. In-unit mortality showed great heterogeneity over the sampling time periods (Fig. 3). Mortality was initially 25.6 per 100 admissions in the two weeks preceding the first PPS but peaked at 32 per 100 admissions before declining to the endline incidence of 3.9 per 100 admissions, representing an overall 85% mortality reduction, though this trend did not reach statistical significance (*p* = 0.064). During the intervention period, there was no change in use of central venous catheters or mechanical ventilation (*p* = 0.72 and *p* = 0.33, respectively) (Supplemental Table 4).

## Discussion

In this quasi-experimental study, we implemented targeted IPC interventions in a NICU with a high burden of BSIs and CROs. There were significant improvements in environmental cleaning practices and hand hygiene compliance concurrent with interventions, suggesting a positive impact from ongoing training efforts. During this time period, colonization with CROs remained largely unchanged, though we did observe downward trends in BSIs. Additionally, although the decrease in mortality did not reach statistical significance, the direction and magnitude of the effect suggest a clinically meaningful downward trend that may warrant further investigation.

The IPC intervention was not designed to be a comprehensive approach to improving IPC or addressing all components of CRO prevention but, rather, was focused on interventions that would be rapid, feasible, and sustainable in a low-resource setting [34]. Although the interventions performed in this NICU were specific to the needs and culture of this unit, we believe that they could be adapted by other NICUs. Additionally, the low baseline rates of hand hygiene and environmental cleaning in this unit are comparable to other reports from Bangladesh, indicating that similar types of interventions may be effective across a wide range of healthcare settings [38–40]. Encouragingly, compliance with hand hygiene and environmental cleaning did not decrease following a 2-month pause due to political instability nor during a 3-month post-intervention audit, suggesting the sustainability of the interventions.

We did not observe a reduction in colonization with MDROs (specifically CROs). Healthy neonates start development of their microbiome at birth; the composition of diverse intestinal flora is influenced by a variety of factors including the maternal microbiome, mode of delivery, and receipt of breast milk [41–45]. These processes are disturbed in premature and sick neonates, which in turn likely predisposes to acquisition of MDROs from the care environment of NICUs or the delivery environment [46–48]. Although we were able to demonstrate improvements in certain IPC domains, these gains alone are unlikely to alter all potential mechanisms of neonatal MDRO colonization. Additionally, we did not assess colonization on admission, so colonization may have been established prior to any exposures in the NICU, precluding us from being able to impact this metric. To complement IPC interventions at the NICU level, effective prevention of colonization with MDROs will require comprehensive and innovative strategies that may encompass perinatal exposures, maternal health, hygiene practices of family members who provide care to neonates, and approaches to support development of a diverse neonatal microbiome.

Consistent with other studies, we also found high rates of antimicrobial administration within this unit, with all neonates receiving antibiotic therapy at some point during their admission [11, 49–54]. As information on reason for antimicrobial administration was not collected, we are not able to draw conclusions on the appropriateness of antimicrobial selection, though it is possible that use of broad-spectrum therapy may have created a fitness advantage to sustain high rates of MDRO colonization. In support of this idea, earlier studies have demonstrated a reduction in MDRO colonization following stewardship interventions [55]. 

This intervention and interpretation of the results are subject to several limitations. Without a comparison group, we cannot draw causal inference about the impact of the interventions on the outcomes or potential external factors contributing to the findings. Additionally, in-depth chart abstraction was not performed during the study period, which prevented our ability to perform detailed analyses into factors such as maternal and birth histories and concurrent medical conditions. We also did not follow neonates longitudinally to monitor outcomes among those who were colonized with CROs versus those who were not. Similarly, we did not assess colonization on admission, so we cannot definitively determine whether infants became colonized because of exposures in this NICU. Furthermore, we only included neonates in the study whose parents/guardians agreed to rectal colonization screening. Although neonates who were not enrolled could have systematically differed from the enrolled infants, we think this possibility is low given that most neonates in the NICU were enrolled during any given PPS. Moreover, we may have overestimated hand hygiene practices as data were collected through direct observation (e.g., Hawthorne effect). Finally, since the intervention period did not include a full calendar year, we are not able to fully assess the impact of seasonality on documented infection or colonization with gram negative organisms [56]. 

In regards to laboratory limitations, it is also likely that we underestimated the true incidence of BSIs that occurred in the population by excluding blood cultures that grew commensal organisms, as commensal organisms can cause true infections in neonates, particularly among those with central venous catheters [57–59]. Additionally, we cannot rule out the possibility that recovery and identification of pathogens from blood cultures may have differed between the lab that used manual methods versus the lab that used automated methods. However, there was already a 75% reduction in BSIs prior to PPS-8 when all blood cultures were processed exclusively by the lab using manual methods.

Given the relatively high uptake of the IPC intervention, it should not be surprising to see a reduced burden of infections as similar impacts have been seen in other settings and, indeed, is why these practices continue to be considered cornerstones in healthcare safety. The notable improvement in hand hygiene and environmental cleaning reiterates that these standard precautions are achievable across a variety of resource levels, aligning with findings from earlier studies in NICUs in LMICs [60–63]. Yet, barriers remain. IPC constraints previously identified in the Bangladesh context extend beyond what was addressed in the current intervention, including insufficient staffing, lack of dedicated IPC personnel, patient overcrowding, and lack of expertise or training, which threaten IPC gains [38, 64]. Healthcare institutions should continue exploring and addressing barriers to consistent implementation of these multimodal IPC strategies.

## Supplementary Information

Below is the link to the electronic supplementary material.

Supplementary Material 1

## Data Availability

All data supporting the findings of this study are available within the paper and its Supplementary Information.

## References

[1] Folgori L, et al. Healthcare-Associated Infections in Pediatric and Neonatal Intensive Care Units: Impact of Underlying Risk Factors and Antimicrobial Resistance on 30-Day Case-Fatality in Italy and Brazil. Infect Control Hosp Epidemiol. 2016;37(11):1302–9. 27511591 10.1017/ice.2016.185

[2] Hooven TA, Polin RA. Healthcare-associated infections in the hospitalized neonate: a review. Early Hum Dev. 2014;90(Suppl 1):S4–6. 24709456 10.1016/S0378-3782(14)70002-7

[3] S B. S. N, and P. M, Immune responses in neonates - PubMed. Expert Rev Clin Immunol 2014 Sep. 10(9). 10.1586/1744666X.2014.942288 PMC4407563 25088080

[4] Collins A, Weitkamp J-H, Wynn JL. *Why are preterm newborns at increased risk of infection?* Archives of disease in childhood. Fetal Neonatal Ed. 2018;103(4):F391–4. 10.1136/archdischild-2017-313595 PMC6013388 29382648

[5] Rosa-Mangeret F, et al. 2.5 Million annual deaths—are neonates in low- and middle-income countries too small to be seen? A bottom-up overview on neonatal morbi-mortality. Trop Med Infect Disease. 2022;7(5):64. 35622691 10.3390/tropicalmed7050064 PMC9148074

[6] Sands K, et al. Characterization of antimicrobial-resistant Gram-negative bacteria that cause neonatal sepsis in seven low- and middle-income countries. Nat Microbiol. 2021;6(4):512. 33782558 10.1038/s41564-021-00870-7 PMC8007471

[7] Johnson J, et al. High burden of bloodstream infections associated with antimicrobial resistance and mortality in the neonatal intensive care unit in Pune, India. Clin Infect Dis. 2021;73(2):271–80. 32421763 10.1093/cid/ciaa554 PMC8282256

[8] Dramowski A, Madide A, Bekker A. Neonatal nosocomial bloodstream infections at a referral hospital in a middle-income country: burden, pathogens, antimicrobial resistance and mortality. Paediatrics Int Child Health. 2015;35(3):265–72. 10.1179/2046905515Y.0000000029 25940506

[9] Milton R, et al. Neonatal sepsis and mortality in low-income and middle-income countries from a facility-based birth cohort: an international multisite prospective observational study. Lancet Global Health. 2022;10(5):e661–72. 35427523 10.1016/S2214-109X(22)00043-2 PMC9023753

[10] Nyma Z et al. Prevalence and associated risk factors of sepsis among neonates admitted into neonatal intensive care units of public hospitals in Dhaka. Cureus. 12(3): p. e7461. 32351840 10.7759/cureus.7461 PMC7188015

[11] Li G, et al. Towards understanding global patterns of antimicrobial use and resistance in neonatal sepsis: insights from the NeoAMR network. Arch Dis Child. 2020;105(1):26–31. 31446393 10.1136/archdischild-2019-316816 PMC6951234

[12] Okeke IN, et al. The scope of the antimicrobial resistance challenge. Lancet. 2024;403(10442):2426–38. 38797176 10.1016/S0140-6736(24)00876-6

[13] Folgori L, et al. The relationship between Gram-negative colonization and bloodstream infections in neonates: a systematic review and meta-analysis. Clin Microbiol Infection: Official Publication Eur Soc Clin Microbiol Infect Dis. 2018;24(3):251–7. 10.1016/j.cmi.2017.08.008 28830807

[14] Schwartz DJ, et al. Gut pathogen colonization precedes bloodstream infection in the neonatal intensive care unit. Sci Transl Med. 2023;15(694):eadg5562. 37134153 10.1126/scitranslmed.adg5562 PMC10259202

[15] Donskey CJ. Does improving surface cleaning and disinfection reduce health care-associated infections? Am J Infect Control. 2013;41(5 Suppl):S12–9. 23465603 10.1016/j.ajic.2012.12.010

[16] Sonpar A, et al. Multimodal strategies for the implementation of infection prevention and control interventions-update of a systematic review for the WHO guidelines on core components of infection prevention and control programmes at the facility level. Clin Microbiol Infect. 2025;31(6):948–57. 39863071 10.1016/j.cmi.2025.01.011

[17] Schreiber PW, et al. The preventable proportion of healthcare-associated infections 2005–2016: Systematic review and meta-analysis. Infect Control Hosp Epidemiol. 2018;39(11):1277–95. 30234463 10.1017/ice.2018.183

[18] Hayden MK, et al. Prevention of colonization and infection by Klebsiella pneumoniae Carbapenemase-producing enterobacteriaceae in long-term acute-care hospitals. Clin Infect Dis. 2015;60(8):1153–61. 25537877 10.1093/cid/ciu1173 PMC8381216

[19] Allegranzi B, Pittet D. Role of hand hygiene in healthcare-associated infection prevention. J Hosp Infect. 2009;73(4):305–15. 19720430 10.1016/j.jhin.2009.04.019

[20] Kochar S, et al. Success of an infection control program to reduce the spread of carbapenem-resistant < i>Klebsiella pneumoniae. Infect Control Hosp Epidemiol. 2009;30(5):447–52. 19301985 10.1086/596734

[21] Popovich KJ, et al. SHEA/IDSA/APIC Practice Recommendation: Strategies to prevent methicillin-resistant Staphylococcus aureus transmission and infection in acute-care hospitals: 2022 Update. Infect Control Hosp Epidemiol. 2023;44(7):1039–67. 37381690 10.1017/ice.2023.102 PMC10369222

[22] Septimus EJ, Schweizer ML. Decolonization in prevention of health care-associated infections. Clin Microbiol Rev. 2016;29(2):201–22. 26817630 10.1128/CMR.00049-15 PMC4786886

[23] Milstone AM, et al. Effect of treating parents colonized With Staphylococcus aureus on transmission to neonates in the intensive care unit: a randomized clinical trial. JAMA. 2020;323(4):319–28. 31886828 10.1001/jama.2019.20785 PMC6990934

[24] Popoola VO, Milstone AM. Decolonization to prevent Staphylococcus aureus transmission and infections in the neonatal intensive care unit. J Perinatol. 2014;34(11):805–10. 25010222 10.1038/jp.2014.128

[25] Hiermandi N, et al. Impact of methicillin-resistant Staphylococcus aureus surveillance and decolonization in the NICU: the Texas children’s hospital experience. Antimicrob Steward Healthc Epidemiol. 2025;5(1):e60. 40026763 10.1017/ash.2025.45 PMC11869050

[26] Gall E, Long A, Hall KK. Chlorhexidine bathing strategies for multidrug-resistant organisms: a summary of recent evidence. J Patient Saf. 2020;16(3S Suppl 1):S16–22. 32809997 10.1097/PTS.0000000000000743 PMC7447168

[27] Miller LG, et al. Decolonization in nursing homes to prevent infection and hospitalization. N Engl J Med. 2023;389(19):1766–77. 37815935 10.1056/NEJMoa2215254 PMC13016440

[28] Climo MW, et al. Effect of daily chlorhexidine bathing on hospital-acquired infection. N Engl J Med. 2013;368(6):533–42. 23388005 10.1056/NEJMoa1113849 PMC5703051

[29] Dramowski A, et al. Impact of 1% chlorhexidine gluconate bathing and emollient application on bacterial pathogen colonization dynamics in hospitalized preterm neonates - A pilot clinical trial. EClinicalMedicine. 2021;37:100946. 34195575 10.1016/j.eclinm.2021.100946 PMC8225683

[30] Sands K, et al. Characterization of antimicrobial-resistant Gram-negative bacteria that cause neonatal sepsis in seven low- and middle-income countries. Nat Microbiol. 2021;6(4):512–23. 33782558 10.1038/s41564-021-00870-7 PMC8007471

[31] Okomo U, et al. Aetiology of invasive bacterial infection and antimicrobial resistance in neonates in sub-Saharan Africa: a systematic review and meta-analysis in line with the STROBE-NI reporting guidelines. Lancet Infect Dis. 2019;19(11):1219–34. 31522858 10.1016/S1473-3099(19)30414-1

[32] Wen SCH, et al. Gram-negative neonatal sepsis in low- and lower-middle-income countries and WHO empirical antibiotic recommendations: a systematic review and meta-analysis. PLoS Med. 2021;18(9):e1003787. 34582466 10.1371/journal.pmed.1003787 PMC8478175

[33] Magiorakos AP, et al. Multidrug-resistant, extensively drug-resistant and pandrug-resistant bacteria: an international expert proposal for interim standard definitions for acquired resistance. Clin Microbiol Infect. 2012;18(3):268–81. 21793988 10.1111/j.1469-0691.2011.03570.x

[34] *Implementation manual to prevent and control the spread of carbapenem-resistant organisms at the national and health care facility level*. 2019, World Health Organization: Geneva.

[35] *Guildeines on core components of infection prevention and control programmes at the national and acute health care facility level*. 2016, World Health Organization: Geneva. 27977095

[36] *Hand Hygiene Technical Reference Manual*. 2009, WHO: Geneva.

[37] National Healthcare Safety Network - Common Commensals 2. /2024; Available from: https://www.cdc.gov/nhsn/xls/master-organism-com-commensals-lists.xlsx

[38] Harun MGD, et al. Hand hygiene compliance and associated factors among healthcare workers in selected tertiary-care hospitals in Bangladesh. J Hosp Infect. 2023;139:220–7. 37516281 10.1016/j.jhin.2023.07.012 PMC11149335

[39] Rimi NA, et al. Infrastructure and contamination of the physical environment in three Bangladeshi hospitals: putting infection control into context. PLoS ONE. 2014;9(2):e89085. 24586516 10.1371/journal.pone.0089085 PMC3929649

[40] Horng LM, et al. Healthcare worker and family caregiver hand hygiene in Bangladeshi healthcare facilities: results from the Bangladesh National Hygiene Baseline Survey. J Hosp Infect. 2016;94(3):286–94. 27665311 10.1016/j.jhin.2016.08.016 PMC5495692

[41] Samarra A, et al. Breastfeeding and early Bifidobacterium-driven microbial colonization shape the infant gut resistome. Nat Commun. 2025;16(1):2025–07. 40603287 10.1038/s41467-025-61154-w PMC12222458

[42] Greenwood C, et al. Early empiric antibiotic use in preterm infants is associated with lower bacterial diversity and higher relative abundance of Enterobacter. J Pediatr. 2014;165(1):23–9. 24529620 10.1016/j.jpeds.2014.01.010 PMC4074569

[43] Yatsunenko TY, et al. Human gut microbiome viewed across age and geography. Nature. 2012;486(7402):222–7. 22699611 10.1038/nature11053 PMC3376388

[44] Obadare TO, et al. Rectal carriage of extended-spectrum β-lactamase-producing Enterobacteriales among neonates admitted into a special care baby unit, southwest Nigeria. Trans R Soc Trop Med Hyg. 2023;117(7):528–35. 36942836 10.1093/trstmh/trad010

[45] Shao Y, et al. Stunted microbiota and opportunistic pathogen colonization in caesarean-section birth. Nature. 2019;574(7776):117–21. 31534227 10.1038/s41586-019-1560-1 PMC6894937

[46] Henderickx JGE, et al. The preterm gut microbiota: an inconspicuous challenge in nutritional neonatal care. Front Cell Infect Microbiol. 2019;9:85. 31001489 10.3389/fcimb.2019.00085 PMC6454191

[47] Palmer C, et al. Development of the human infant intestinal microbiota. PLoS Biol. 2007;5(7):e177. 17594176 10.1371/journal.pbio.0050177 PMC1896187

[48] Carvalho MJ, et al. Antibiotic resistance genes in the gut microbiota of mothers and linked neonates with or without sepsis from low- and middle-income countries. Nat Microbiol. 2022;7(9):1337–47. 35927336 10.1038/s41564-022-01184-y PMC9417982

[49] Uzan-Yulzari A, et al. Neonatal antibiotic exposure impairs child growth during the first six years of life by perturbing intestinal microbial colonization. Nat Commun. 2021;12(1):443. 33500411 10.1038/s41467-020-20495-4 PMC7838415

[50] Schulman J, et al. Newborn antibiotic exposures and association with proven bloodstream infection. Pediatrics. 2019;144(5):e20191105. 31641017 10.1542/peds.2019-1105

[51] Girardi A, et al. Pattern of drug use among preterm neonates: results from an Italian neonatal intensive care unit. Ital J Pediatr. 2017;43(1):37. 28412957 10.1186/s13052-017-0354-z PMC5392975

[52] Persaud RR, et al. Perinatal antibiotic exposure of neonates in Canada and associated risk factors: a population-based study. J Matern Fetal Neonatal Med. 2015;28(10):1190–5. 25053193 10.3109/14767058.2014.947578

[53] Solomon S, et al. Prevalence and risk factors for antimicrobial resistance among newborns with gram-negative sepsis. PLoS ONE. 2021;16(8):e0255410. 34343185 10.1371/journal.pone.0255410 PMC8330902

[54] J, J., et al., *Risk Factors for Health Care-Associated Bloodstream Infections in NICUs - PubMed.* JAMA network open, 03/03/2025. 8(3). 10.1001/jamanetworkopen.2025.1821 PMC11937935 40131271

[55] Calil R, et al. Reduction in colonization and nosocomial infection by multiresistant bacteria in a neonatal unit after institution of educational measures and restriction in the use of cephalosporins. Am J Infect Control. 2001;29(3):133–8. 11391273 10.1067/mic.2001.114223

[56] Richet H. Seasonality in Gram-negative and healthcare-associated infections. Clin Microbiol Infect. 2012;18(10):934–40. 22784293 10.1111/j.1469-0691.2012.03954.x

[57] Healy CM, et al. Distinguishing true coagulase-negative Staphylococcus infections from contaminants in the neonatal intensive care unit. J Perinatol. 2013;33(1):52–8. 22499081 10.1038/jp.2012.36

[58] Zelellw DA, et al. A systemic review and meta‐analysis of the leading pathogens causing neonatal sepsis in developing countries. Biomed Res Int. 2021;2021(1):6626983. 34195273 10.1155/2021/6626983 PMC8203353

[59] Samarra A et al. 2024 Unravelling the evolutionary dynamics of antibiotic resistance genes in the infant gut microbiota during the first four months of life. Annals of clinical microbiology and antimicrobials, 23(1), p.72. 39138497 10.1186/s12941-024-00725-z PMC11323388

[60] Johnson J, et al. Implementation of the Comprehensive Unit-Based Safety Program to Improve Infection Prevention and Control Practices in Four Neonatal Intensive Care Units in Pune, India. Front Pead. 2022. . 10.3389/fped.2021.794637 PMC8772032 35071137

[61] Amaan A, K Dey S, Zahan K. Improvement of hand hygiene practices among the healthcare workers in a neonatal intensive care unit. Can J Infect Dis Med Microbiol = J Canadien Des Maladies Infectieuses Et De La Microbiologie Medicale. 2022;2022:p7688778. 10.1155/2022/7688778 PMC9252715 35795864

[62] Donovan EF, et al. The investment case for preventing NICU-associated infections. Am J Perinatol. 2013;30(3):179–84. 22836823 10.1055/s-0032-1322516 PMC3789586

[63] Dramowski A, et al. NeoCLEAN: a multimodal strategy to enhance environmental cleaning in a resource-limited neonatal unit. Antimicrob Resist Infect Control. 2021;10:35. 33579364 10.1186/s13756-021-00905-y PMC7881651

[64] Harun MGD, et al. Infection prevention and control in tertiary care hospitals of Bangladesh: results from WHO infection prevention and control assessment framework (IPCAF). Antimicrob Resist Infect Control. 2022;11(1):125. 36203207 10.1186/s13756-022-01161-4 PMC9535892

