# Supplementary material for: Impact of targeted infection prevention and control interventions on bloodstream infections and colonization with carbapenem-resistant organisms among hospitalized neonates, Bangladesh, 2023–2024
Source: Antimicrob Resist Infect Control. 2026 May 20;15:85. doi: 10.1186/s13756-026-01765-0 (PMC13242672; doi:10.1186/s13756-026-01765-0)
Supplement: Supplementary file 1 — Supplementary Material 1 [file 13756_2026_1765_MOESM1_ESM.docx]

**Supplemental Figure 1. IPC instructional posters drawn by a local Bangladeshi artist.**

**
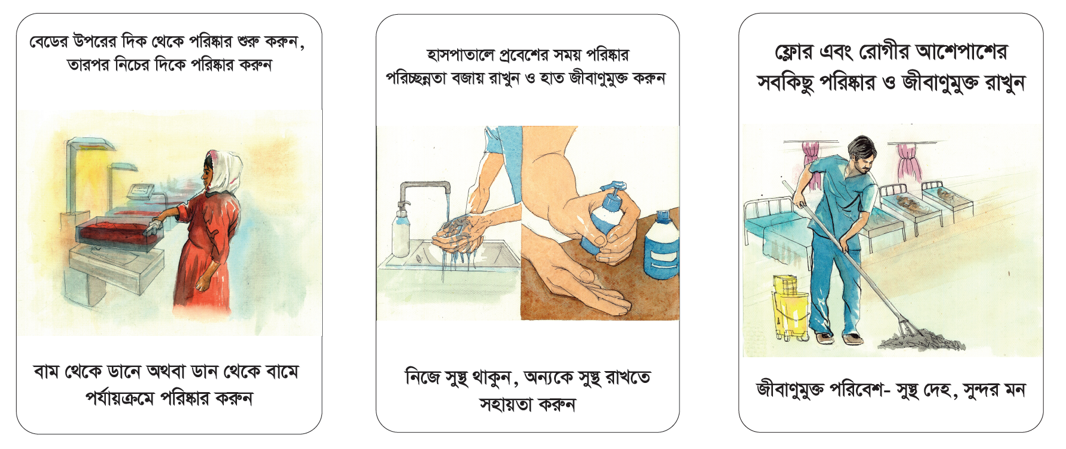
**

Image credit: xx

**Supplemental Table 1. Surfaces and equipment included in environmental cleaning assessments in a neonatal intensive care unit in Bangladesh**

| **Patient zone surfaces** | **Common surfaces** | **Shared equipment** |
| --- | --- | --- |
| Crib rail | Nurses’ charting table or chairs | Portable or wall-mounted suction machines |
| Crib mattress | Nurses’ station telephone | Nebulizer machine |
| Crib storage drawers | Nurses’ station microphone | Stethoscopes |
| Crib shelf | Sink faucet | Thermometers |
| Radiant warmer stand | Doorknobs | Xray machine |
| IV pump control or IV stand | Medication and supply carts | Weight scales |
| Vital signs monitor | Light switches | Ultrasound machine |
| Phototherapy light |  |  |
| Oxygen facemask |  |  |
| Ventilator panel |  |  |

**Supplemental Table 2.** IPC metrics, colonization prevalence, BSIs, and mortality in a NICU in Bangladesh during an IPC intervention, February-September 2024

|  | **PPS 1** | **PPS 2** | **PPS 3** | **PPS 4** | **PPS 5** | **PPS 6** | **PPS 7** | **PPS 8** | **PPS 9** | **PPS 10** | **PPS 11** | **PPS 12** |
| --- | --- | --- | --- | --- | --- | --- | --- | --- | --- | --- | --- | --- |
| **PPS date** | 2/12 | 2/27-2/28 | 3/13-3/14 | 3/27-3/28 | 4/8 | 4/22-4/23 | 5/8-5/9 | 5/25-5/26 | 6/10-6/11 | 9/1 | 9/15 | 9/29 |
| **Dates included (for admissions, BSIs, deaths)** | 2/1-2/12 | 2/13-2/28 | 2/29-3/14 | 3/15-3/28 | 3/29-4/8 | 4/9-4/23 | 4/24-5/9 | 5/10-5/26 | 5/27-6/11 | 8/1-8/31 | 9/1-9/14 | 9/15-9/30 |
| **HH/EC assessment dates** | 2/1, 2/7 | 2/14, 2/20, 2/25 | 3/2, 3/10 | 3/18, 3/27 | 4/2 | 4/18 | 5/2 | 5/14 | 6/1 | 8/1, 8/20 | 9/1 | 9/15 |
| **HH compliance** | 37% | 48% | 44% | 55% | 57% | 64% | 67% | 65% | 64% | 68% | 71% | 69% |
| **EC compliance** | 42% | 46% | 50% | 61% | 67% | 56% | 67% | 73% | 67% | 80% | 80% | 87% |
| **Admissions** | 39 | 46 | 57 | 34 | 48 | 54 | 57 | 61 | 69 | 117 | 47 | 77 |
| **PPS enrollment** | 30 | 33 | 38 | 23 | 28 | 31 | 30 | 28 | 30 | 28 | 32 | 32 |
| **BSIs** | 11 | 9 | 6 | 1 | 6 | 4 | 4 | 2 | 0 | 1 | 5 | 3 |
| **Deaths** | 10 | 5 | 7 | 11 | 12 | 12 | 14 | 9 | 10 | 24 | 4 | 3 |
| **CRO colonization** | 87% | 91% | 74% | 87% | 86% | 87% | 67% | 68% | 80% | 64% | 94% | 81% |
| **CRE colonization** | 77% | 58% | 58% | 78% | 54% | 74% | 37% | 39% | 70% | 46% | 66% | 69% |
| **CR-Kp colonization** | 63% | 48% | 42% | 78% | 54% | 55% | 27% | 36% | 50% | 46% | 50% | 59% |
| **BSIs/100 admissions** | 28.2 | 19.6 | 10.5 | 2.9 | 12.5 | 7.4 | 7.0 | 3.3 | 0.0 | 0.9 | 10.6 | 3.9 |
| **Deaths/100 admissions** | 25.6 | 10.9 | 12.3 | 32.4 | 25.0 | 22.2 | 24.6 | 14.8 | 14.5 | 20.5 | 8.5 | 3.9 |

*IPC = infection prevention and control, BSI = bloodstream infection, NICU = neonatal intensive care unit, PPS = point prevalence survey, HH = hand hygiene, EC = environmental cleaning, CRO = carbapenem-resistant organisms, CRE = carbapenem resistant Enterobacterales, CR-Kp = carbapenem-resistant* K. pneumoniae*. The intervention was paused for 2 months during July-August because of political instability (indicated by the double line between PPS 9 and PPS 10).*

**Supplemental Figure 2.** IPC metrics, colonization prevalence, BSIs, and mortality in a NICU in Bangladesh during an IPC intervention, February-September 2024


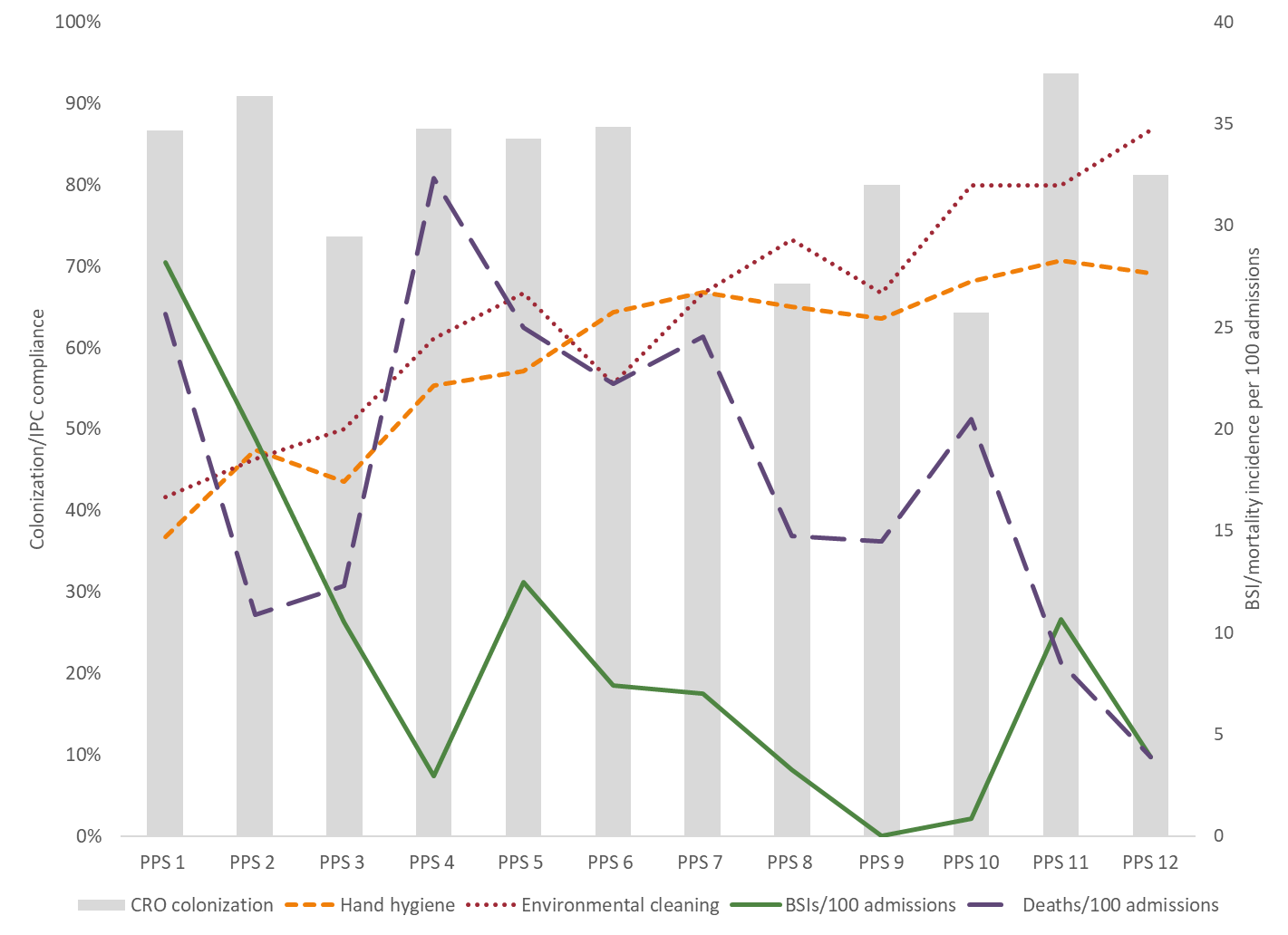


*IPC = infection prevention and control, BSI = bloodstream infection, NICU = neonatal intensive care unit, PPS = point prevalence survey, CRO = carbapenem-resistant organism. The intervention was paused for 2 months during July-August between PPS-9 and PPS-10 because of political instability.*

Supplemental Table 3. Pathogens recovered from blood cultures among neonates with suspected infections in a Bangladesh NICU, February-September 2024

| Organism name | Frequency |
| --- | --- |
| *A. baumannii* | 7 |
| *B. cepacia* | 7 |
| *C. albicans* | 3 |
| *C. ciferrii* | 2 |
| *C. pelliculosa* | 3 |
| *C. indologenes* | 1 |
| *C. parapsilosis* | 1 |
| *Candida spp.* | 8 |
| *Enterococcus spp.* | 1 |
| *E. coli* | 1 |
| *K. pneumoniae* | 3 |
| *K. ohmeri* | 1 |
| *Pseudomonas spp.* | 6 |
| *S. fonticola* | 6 |
| *S. marcescens* | 1 |
| Unclassified | 1 |
| Total | 52 |

List excludes common commensals.

Supplemental Table 4. Proportion of neonates with peripheral IV, central venous line (umbilical catheter), and mechanical ventilation by PPS in a Bangladesh NICU, February-September 2024

| **PPS** | **Peripheral IV** | **Central venous line** | **Mechanical ventilation** |
| --- | --- | --- | --- |
| PPS 1 | 23 (76.7%) | 4 (13.3%) | 1 (3.3%) |
| PPS 2 | 29 (87.9%) | 0 (0%) | 2 (6.1%) |
| PPS 3 | 33 (86.8%) | 1 (2.6%) | 2 (5.3%) |
| PPS 4 | 14 (60.9%) | 1 (4.3%) | 0 (0%) |
| PPS 5 | 26 (92.9%) | 2 (7.1%) | 1 (3.6%) |
| PPS 6 | 28 (90.3%) | 3 (9.7%) | 1 (3.2%) |
| PPS 7 | 28 (93.3%) | 2 (6.7%) | 1 (3.3%) |
| PPS 8 | 21 (75.0%) | 5 (17.9%) | 1 (3.6%) |
| PPS 9 | 26 (86.7%) | 4 (13.3%) | 2 (6.7%) |
| PPS 10 | 25 (89.3%) | 1 (3.6%) | 0 (0%) |
| PPS 11 | 28 (87.5%) | 0 (0%) | 2 (6.3%) |
| PPS 12 | 27 (84.4%) | 0 (0%) | 3 (9.4%) |

*IPC = infection prevention and control, PPS = point prevalence survey, NICU = neonatal intensive care unit.*
